# Early Onset Alzheimer's Disease and Oxidative Stress

**DOI:** 10.1155/2014/375968

**Published:** 2014-01-14

**Authors:** Marco Antonio Meraz-Ríos, Diana Franco-Bocanegra, Danira Toral Rios, Victoria Campos-Peña

**Affiliations:** ^1^Departamento de Biomedicina Molecular, Centro de Investigación y de Estudios Avanzados del Instituto Politécnico Nacional, Instituto Politécnico Nacional 2508, 07360 Mexico City, Mexico; ^2^Posgrado en Ciencias Biológicas, Universidad Nacional Autónoma de México, Universidad 3000, Coyoacan, 04510 Mexico City, Mexico; ^3^Departamento de Fisiología Biofísica y Neurociencias, Centro de Investigación y de Estudios Avanzados del Instituto Politécnico Nacional, Instituto Politécnico Nacional 2508, 07360 Mexico City, Mexico; ^4^Laboratorio Experimental de Enfermedades Neurodegenerativas, Instituto Nacional de Neurología y Neurocirugía Manuel Velasco Suárez, Insurgentes Sur 3877, 14269 Mexico City, Mexico

## Abstract

Alzheimer's disease (AD) is the most common cause of dementia in elderly adults. It is estimated that 10% of the world's population aged more than 60–65 years could currently be affected by AD, and that in the next 20 years, there could be more than 30 million people affected by this pathology. One of the great challenges in this regard is that AD is not just a scientific problem; it is associated with major psychosocial and ethical dilemmas and has a negative impact on national economies. The neurodegenerative process that occurs in AD involves a specific nervous cell dysfunction, which leads to neuronal death. Mutations in APP, PS1, and PS2 genes are causes for early onset AD. Several animal models have demonstrated that alterations in these proteins are able to induce oxidative damage, which in turn favors the development of AD. This paper provides a review of many, although not all, of the mutations present in patients with familial Alzheimer's disease and the association between some of these mutations with both oxidative damage and the development of the pathology.

## 1. Introduction

Brain requires a high consumption of oxygen to generate adenosine triphosphate (ATP). It is known that oxygen metabolism in the mitochondria, endoplasmic reticulum (ER), and peroxisomes generates oxidant agents known as free radicals [1, 2], small molecules with unpaired electron that includes the oxygen reactive species (ROS) like hydroxyl radical (OH^−^), superoxide radical (O_2_^−^), the reactive nitrogen species (RNS), and nitric oxide (NO^•^). These molecules show high reactivity with macromolecules [3] and have an important biological function as signaling molecules [4]. However the interaction of these agents and nonradical oxidants with membrane lipids, proteins, and DNA also could be conducted to cellular senescence. This oxidative damage is catalyzed by the presence of trace elements Fe, Cu or both [5].

As part of evolution, organisms have developed enzymatic and nonenzymatic antioxidants mechanism to counteract oxidative damage, which act removing free radicals, scavenging ROS/RNS or their precursors and binding trace elements [1]. The antioxidant enzymes are superoxide dismutase (SOD), catalase, and glutathione peroxidase (GPx). The nonenzymatic antioxidants group is composed of the natural molecules glutathione (GSH) and the reduced form of nicotinamide adenine dinucleotide phosphate (NADPH), and compounds like ascorbic and lipoic acid, polyphenols and carotenoids dietary derived [6].

However, an imbalance of oxidants and antioxidants agents could generate oxidative stress, which results in a damage of macromolecules and disruption of reduction/oxidation (redox) signaling [7]. Mitochondrial dysfunction, excitotoxicity, and oxidative stress are common pathological conditions of neurodegenerative diseases such as Parkinson's disease, Multiple Sclerosis, Amyotrophic Lateral Sclerosis, and Alzheimer's disease (AD) [8, 9].

AD is a disorder of the central nervous system (CNS) that results in generalized brain atrophy. Clinically, AD is characterized by the gradual and progressive loss of memory and other cognitive functions, such as the ability to solve everyday problems and emotional control [10–12]. Conventionally, AD is divided in two subtypes, depending on the age of onset: familial cases and sporadic cases. Familial AD (FAD), which accounts for only 5–10% of all AD cases, exhibit an autosomal dominant form of inherited mutation in the amyloid precursor protein gene and the presenilin 1 or 2 genes and are characterized by an age of onset prior to 55 years old (early onset AD (EOAD)). Sporadic cases account for 90–95% of all AD cases and usually present a later age of onset (≥65 years). These cases do not show the familial aggregation associated with the early development of the disease and are known as late onset AD (LOAD). Twin studies provide insight into the relative contributions of genetic and environmental influences on AD and other types of dementia [13–15]. It has been observed that among patients who develop LOAD, approximately 40–65% present apolipoprotein E allele 4 (APOEe4) as an indirect genetic agent [16–19]. However, the presence of APOEe4 as a genetic risk factor is not enough for developing the disease [20, 21]. Histopathologically, AD is defined by the presence of two specific features: neuritic plaques (NPs) and neurofibrillary tangles (NFT) [22–24]. *In vitro* and *in vivo* data now support the notion that the accumulation of both A*β*-containing senile plaques and tau-containing neurofibrillary tangles (NFTs) in the brain can directly or indirectly cause free radical-induced stress. Mutations in APP and PS can increase reactive oxygen species (ROS) production and generate mitochondrial damage which in turn favors the neurodegenerative process observed in AD. This paper reviews the general characteristics of FAD, the mutations carried by APP and PS in transgenic mouse models, and their role in oxidative damage.

## 2. Neurofibrillary Tangles 

NFTs are intracellular deposits of paired helical filaments (PHFs). NFT density in AD patients' brain is closely related to dementia severity [25, 26]. In these filaments, the most important molecular marker is tau, a microtubule-associated protein. The gene that encodes this protein is located on chromosome 17 [27]. In the adult human brain, six tau isoforms are produced via alternative splicing of exons 2, 3, and 10. When exon 10 is excluded, the result is a protein with three repeats of the microtubule-binding domain (3RMBD). When exon 10 is included, a fourth microtubule binding domain is added to generate four-repeat tau (4RMBD) [28–30]. Tau is a highly soluble protein that is natively unfolded and does not show an apparent secondary structure [31, 32] due to high proline and glycine content in its primary structure. However, under pathological conditions, tau tends to self-assemble into insoluble filament structures [33]. This protein is implicated in neurodegeneration in many disorders, such as AD, progressive supranuclear palsy (PSP), corticobasal degeneration (CBD), Pick's disease (PiD), Down's syndrome (DS), postencephalitic Parkinsonism, and Niemann-Pick disease [34–36]. Mutations in tau gene cause frontotemporal dementia with Parkinsonism linked to chromosome 17 (FTDP-17) [37–40]; however, AD pathology is not related to mutations in the tau gene.

## 3. Neuritic Plaques

**Figure 1:**
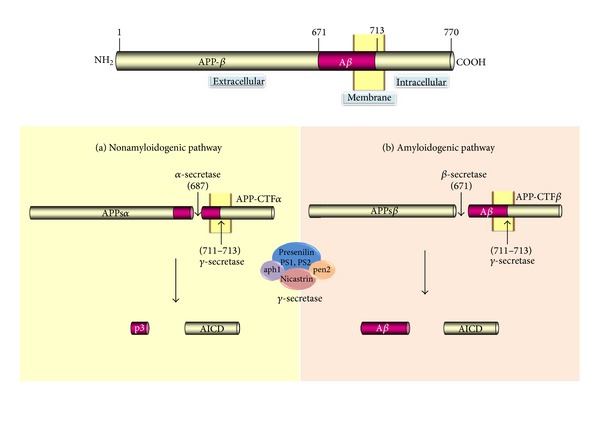
APP*β* processing. the APP is an integral membrane protein and is sequentially processed by the three proteases *α*-, *β*-, and *γ*-secretase. The nonamyloidogenic pathway involves the *α*-secretase, which made the cut at the middle portion of the fragment corresponding to the amyloid sequence, preventing the amyloid peptides generation. The amyloidogenic pathway involves *β*-secretase, leading to the formation of C-terminal fragments (CTFs) that are subsequently cleaved by the “**γ**-secretase-complex” which is responsible for the formation of A**β** (40 or 42 amino acids in length) and the A**β**PP intracellular domain peptide (AICD) of 58 or 56 amino acids.

NPs are one of the stereotypical markers of AD; they are spherical extracellular deposits, 10–100 *μ*m in diameter, containing a fibrillary core surrounded by microglia, reactive astrocytes, and dystrophic neurites from degenerating neuronal processes [41]. The main component of NPs is amyloid-*β* (A*β*), a 39–42 amino acids peptide [42, 43] that originates as a normal secretory product derived from amyloid precursor protein (APP) [44]. The primary function of APP remains unknown, although it has been proposed that it could participate as a growth factor in cultured fibroblasts [45], play roles in cell adhesion [46], intraneuronal calcium regulation [47], and neural plasticity [48], and act as a synapse formation regulator [49]. APP undergoes two types of proteolytic processing, resulting in the generation of two distinct classes of peptides with different biological roles [45]: (a) soluble APP (*α*APPs) via proteolytic processing by *α*- and *γ*-secretase and (b) amyloid-*β* peptides via proteolytic processing of APP by *β*- and *γ*-secretase (Figure 1). *γ*-secretase is a protein complex consisting of presenilin 1 (PS1)/presenilin 2 (PS2), nicastrin (NCT), anterior pharynx-defective 1 (APH-1), and presenilin enhancer 2 (PEN-2) [50–53].

## 4. Amyloid-***β*** and Oxidative Stress

In AD, A*β* peptides of 40 and 42 amino acids acquire a *β*-sheet structure, which is proaggregator and leads to the formation of dimers, oligomers, insoluble fibers, and NP formation [54, 55]. Oligomers represent the most toxic aggregation stage of A*β*, because they promote excitotoxicity by interacting with glutamate receptors, endoplasmic reticulum stress, mitochondrial dysfunction, altered acetyl-cholinergic neurotransmission, inflammation, and oxidative stress [56].

The transition metals, Cu^2+^, Zn^2+^, and Fe^3+^, are altered in AD brain and they have been involved with A*β* aggregation and oxidative damage [57]. In particular, A*β* has three histidine residues at positions 6, 13, and 14 for metals coordination. A*β* catalyses the reduction of Cu^2+^ and Fe^3+^ and generates H_2_O_2_, which is converted to OH^•^ in the presence of the metals Cu^1+^ and Fe^2+^; the generation of this reactive species leads to the formation of proapoptotic lipid peroxidation (LPO) products, such as 4-hydroxynonenal (HNE) [58, 59]. In contrast, an *in vitro* study showed that Zn^2+^ quenched A*β*-Cu^2+^ complexes, promoting an antioxidant function [60].

Another important amyloid residue that is related with the oxidative stress is the Methionine 35 (Met^35^). The expression of human A*β* 1–42 in *Caenorhabditis elegans *(*C. elegans*) promoted an increase of protein oxidation levels, compared with the *C. elegans* transgenic line CL3115 that express a substitution of Met^35^ by a Cysteine (replacement of the S atom in Met by CH_2_) [61]. In addition, the J20 transgenic mouse with human APP containing Swedish (KM670/671NL) and Indiana (V717F) mutations present elevated A*β* deposition and increased oxidative stress in the brain around 5–7 months old. Introduction of M631L mutation to APP (corresponding to the Met^35^ residue of A*β*) in J20 mouse resulted in no oxidative stress in brain at 9 months old [62]. The mechanism of Met^35^ leading oxidative damage involves the A*β* binding to Cu^2+^; this reaction generates H_2_O_2_ that could cause the oxidative modification of the sulphur atom of Met^35^ generating sulphuryl free radical. This species favors ROS formation in the lipid bilayer, promoting LPO and membrane protein oxidation [63]. It has been documented that the induction of methionine-sulfoxide reductase prevents the oxidation of Met^35^ residue, suggesting that this enzyme could be a therapeutic target in order to decrease the oxidative activity of A*β* aggregates [64]. Despite this, A*β* would promote oxidative stress through other indirect mechanisms. The A*β* accumulation in parenchyma and blood vessels causes microglial migration and promotes acute and chronic inflammatory responses against the aggregates, thus inducing the production of proinflammatory cytokines, prostaglandins, NO, and ROS, which eventually could promote neuronal death [65]. Also, A*β* oligomers activate the N-methyl-D-aspartate receptor (NMDA-R), leading to a rapid influx of calcium, which promote ROS generation from the NADPH oxidase. These effects are counteracted by memantine, an open channel NMDA-R antagonist prescribed as a memory-preserving drug for AD patients [66, 67]. Finally, the A*β* accumulation in the mitochondria is conducted to morphological alterations, and also a functional impairment including a decrease of ATP, increasing ROS generation, and breaking membrane potential that leads to cellular apoptosis [68, 69].

The mechanisms of A*β* to generate oxidative stress take a high impact on the fast progression of EOAD, because all germline mutations are conducted to an increase of A*β* production and aggregation. Immunotherapy with anti-A*β* antibodies has been tested in transgenic mouse model, resulting in a prevention of synaptotoxicity of A*β* aggregates [70].

## 5. Early Onset Alzheimer's Disease

FAD or EOAD accounts for less than 10% of cases and is associated with mutations in proteins such as PS1, PS2, and APP. These mutations are closely related to the early onset of the disease, with a high penetrance being observed among mutation carriers [71–79]. Currently, more than 200 distinct disease-causing mutations have been identified across these genes, which exhibit an autosomal dominant disease-transmission pattern.

## 6. APP Mutations

**Figure 2:**
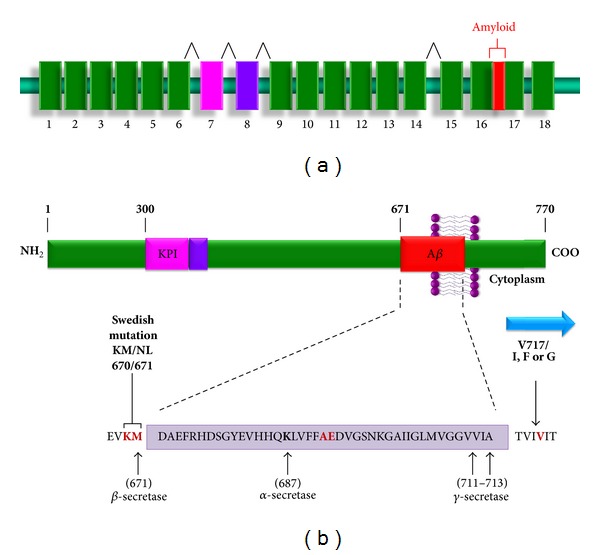
Human APP gene structure. (a) The APP gene consisting of 18 exons is located on chromosome 21 (21q21.2-3) and is alternatively spliced into several products, named according to their length in amino acids (i.e., APP695, APP714, APP751, APP770, and APP563) that are expressed differentially by tissue type. The region encoding the amyloid sequence comprises part of exons 16 and 17 (red box). (b) APP is a member of a family of conserved type I membrane proteins and consists of a large extracellular domain, a hydrophobic transmembrane domain, and a short cytoplasmic carboxyl terminus. Some isoforms contain a domain homologous to the Kunitz-type serine protease inhibitors (KPI) in the extracellular sequences (pink box). Amyloid sequence contains 40- and 43-amino-acid residues that extend from the ectodomain into the transmembrane domain of the protein. The A*β* sequence lies partially outside the cell membrane (amino acids 1–17 of A*β*) and the some identified mutations in the protein are indicated in bold.

APP is a type I integral membrane glycoprotein that resembles a signal-transduction receptor [44] (Figure 2). The APP gene has been mapped to chromosome 21q21 and consists of 18 exons. Alternative splicing generates several isoforms of this gene, which are designated according to amino acid length: APP563, APP695, APP714, APP751, and APP770. In the CNS, the only isoforms present are APP695, APP714, APP751, and APP770, with APP695 being mainly expressed in neurons. To date, approximately 36 different missense mutations in the APP gene have been identified among 85 families (Table 1). Most of these mutations are located in exons 16-17, in the transmembrane domain, where the sites recognized by the *α*-, *β*-, and *γ*-secretases are found (Figure 2(b)). These mutations alter the processing of the protein and cause the accumulation of A*β*42 fragments by decreasing A*β*40 peptide levels or increasing A*β*42 production [74, 78].

Mutations in APP linked to EOAD include the Dutch (E693Q) [80], London (V717I) [74], Indiana (V717F) [77], Swedish (K670N/M671L) [81], Florida (I716V) [82], Iowa (D694N) [83], and Arctic (E693G) [84] mutations. The major mutations in APP include the Swedish double mutation (APPSW, APPK670N, and M671L) and the London mutation (V717I). In 1991, Goate et al. identified a missense mutation in the gene encoding APP that segregates with AD. This mutation is located in exon 17 in part of the sequence encoding the A*β* peptide and leads to a valine to isoleucine change at amino acid 717 (V717I) [74], corresponding to the transmembrane domain near the *γ*-secretase cleavage site. The Swedish mutation, which is located just outside the N-terminus of the A*β* domain of APP, favors *β*-secretase cleavage and it is associated with increased levels and deposition of A*β*42 in the brains of AD patients [85, 86].

## 7. APP Mutations and Oxidative Stress

The presence of APP mutations in EOAD leads to increased levels of A*β*, which may result in mitochondrial dysfunction and augmented ROS levels, thus increasing oxidative damage. A role of A*β* causing mitochondrial dysfunction has been extensively reported. It is known that A*β* is able to decrease mitochondrial complexes I and IV activity, leading to electron transport chain and oxidative phosphorylation dysfunction, which in turn causes adenosine triphosphate (ATP) depletion [87, 88]. Additionally, A*β* stimulates mitochondrial permeability transition pore opening, thus disturbing mitochondrial ion balance [89]. A*β* has been also linked with mitochondrial dynamics dysfunction [90]. All this mitochondrial alterations might in turn lead to an increase in ROS production and consequently enhance oxidative stress.

Transgenic animal models that overexpress mutant APP have been useful in the assessment of the oxidative damage that occurs when A*β* levels increase. This was observed in isolated mitochondria taken from transgenic mice expressing a double Swedish/London mutation of APP. The results showed both very marked mitochondrial dysfunction and reduced ATP-levels in adult APP mice. These alterations were present after three months, at which point amyloid intracellular levels were noted to have increased, while no extracellular A*β* deposits were present. Mitochondrial dysfunction was associated with higher levels of ROS, with a decreased Bcl-xL/Bax ratio and a reduction of mitochondrial complex IV activity. There is evidence that oxidative stress might cause an upregulation of Bax [91]. This increase in the activity of Bax and other proapoptotic members of the Bcl-2 family could be playing a role in enhancing the massive neuronal loss observed in AD patients. [92].

Isoprostanes (iPs) are specific and sensitive markers of *in vivo* lipid peroxidation (LPO). Tg2576 mice, which develop A*β* brain deposits due to the overexpression of a transgene with a double Swedish mutation (APPswe), were used to determine levels of iPs and LPO. Urine, plasma, and brain tissues were collected from both Tg2576 and wild-type (WT) animals at different ages, starting at four months old and continuing until eighteen months old. The results showed that, compared with WT mice, iP levels increased at eight months old in Tg2576 mice and preceded the onset of A*β* deposition in the CNS [93]. It has been shown that LPO products, such as HNE are diffusible and highly reactive with other biomolecules and thus are neurotoxic. The results obtained in this AD model are coincident with previous reports that show that HNE levels are increased in the AD brain [94].

In this way, superoxide dismutase (SOD) and glutathione peroxidase (GPx) activities are found to increase in cortical tissue, while the level of nitric oxide and reactive nitrogen species showed peak values around nine months old [95]. These results might suggest that in the Tg2576 mouse model, LPO and the elevation of antioxidants precede amyloid plaque formation. Notably, the ages at which these oxidative stress peaks occur are coincident with the ages at which these mice begin to present impaired cognitive performance with respect to control mice, leaving open the possibility that oxidative stress could account for cognitive impairment in this model.

It has also been observed that mitochondrial A*β* accumulation increased around four months-old (before plaque formation) in transgenic APP mice expressing both APP V717/F and the APP Swedish mutation, suggesting an intracellular A*β* toxicity cascade [96].

Another FAD mouse model features Thy1-APP751SL mice, which are made transgenic by the 751 amino acid form of APP are used with the Swedish and London mutations under the control of the promoter Thy1. These mice overexpress APP and develop both high levels of A*β* and plaque formation at six months old. HNE levels were significantly higher in twelve months old animals, while the overexpression of APP led to reduced Cu/Zn-SOD activity at three and twelve months of age and had a more pronounced effect on twelve months old animals [97, 98].

## 8. Presenilin Mutation

Most FAD cases are associated with mutations in PS1 or PS2 [71, 76, 99]. These mutations are autosomal dominant and highly penetrant. Presenilins are expressed in several tissues and in the brain, but they are expressed mainly in neurons [75]. Presenilins localize in the endoplasmic reticulum (ER), Golgi apparatus, endosomes, lysosomes, phagosomes, plasma membranes, and mitochondria [100–102]. These proteins undergo endoproteolytic processes, generating stable N- and C-terminal fragments (NTF and CTF, resp.). These fragments interact with other proteins to form a macromolecular complex with *γ*-secretase activity, which is responsible for the intramembranal proteolysis of APP and other proteins [51, 85, 103–106]. Both PS1 and PS2 possess the conserved aspartate residues required for *γ*-secretase activity [107]. In addition to this function, presenilins directly or indirectly regulate the trafficking and metabolism of select membrane proteins in neurons [108]. Studies in several models have shown that presenilins play roles in synaptic function [109, 110], learning and memory [111], neuronal survival in the adult brain, regulation of calcium homeostasis [112, 113], and presynaptic neurotransmitter release [114]. PS1 function loss has been reported to inhibit normal migratory neuronal trajectories during neurodevelopment [115]. Mutations in PS1 and PS2 induce A*β* overproduction, apparently by increasing *γ*-secretase activity [116–120], which is the final step in amyloid peptide formation. Although transgenic mice with a single mutation in either PS1 or PS2 do not form plaques, they exhibit a number of pathological features, including age-related neuronal and synaptic loss as well as vascular pathology.

## 9. Presenilin 1

**Figure 3:**
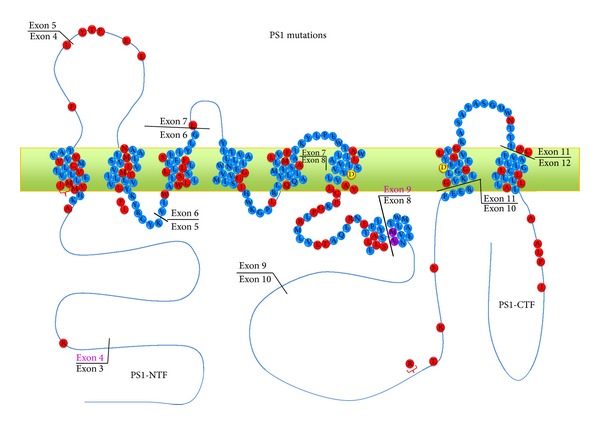
Schematic representation of Presenilin 1. Presenilins are membrane proteins that form the catalytic core of the *γ*-secretase complex. The PSEN1 gene is located on chromosome 14q24.2 and comprises 12 exons. PS1 is an integral membrane protein with eight transmembrane domains and a hydrophilic domain between domains 6 and 7. Two aspartate residues in transmembrane domains, (TMs) 6 and 7 constituting the catalytic site. To date, more than 185 mutations in PSEN1 have been described in 405 families all of which are related to the appearance of the disease at younger ages. Although mutations are found throughout the protein, most are located in the transmembrane region.

The PS1 gene is located on chromosome 14q24.2 and comprises 12 exons. The open reading frame is encoded in exons 3–12 and generates 467 amino acids length protein. PS1 is an integral membrane protein with eight transmembrane domains and a hydrophilic domain between domains 6 and 7. To date, more than 185 mutations in PS1 have been described in 405 families (http://www.molgen.ua.ac.be/ADmutations/), all of which are related to a disease onset at younger ages than sporadic AD cases [121, 122]. Although mutations are found throughout the protein, most are located in the transmembrane region (Figure 3). As shown by Shen et al. in 1997, PS1-knockout mice are not viable, and the results obtained in this study showed that PS1 is required for proper formation of the axial skeleton and for normal neurogenesis in mice and that it plays an important role in neuronal viability in specific brain subregions [123]. Selective expression of mutant PS1 in mice causes a gain of deleterious function that increases the amount of A*β*42 in the brain [73]. This effect was detectable as early as 2–4 months old, and different PS1 mutations were found to have differential effects on A*β* generation [71, 124]. Transgenic mice carrying the M233T/L235P knock-in (KI) mutations in PS1 and human APP show extensive neuronal loss (>50%) in the CA1/2 hippocampal pyramidal cell layer at 10 months old, which is correlated with intraneuronal amyloid accumulation, strong reactive astrogliosis, and neuronal loss [125]. Likewise, it has been reported that transient intraneuronal amyloid accumulation is correlated with neuronal loss in the frontal cortex of APP/PS1KI mice, rather than extracellular plaque pathology [126]. Breyhan and coworkers demonstrated that intraneuronal accumulation of A*β* peptides, together with oligomeric and fibrillary accumulation species, coincided with 30% of neuronal loss in the CA1 region, 18% of hippocampus atrophy and a severe reduction of synaptic plasticity [127]. In addition to its role in *γ*-secretase activity, PS1 appears to modulate glycogen synthase kinase-3*β* (GSK-3*β*) activity and the release of kinesin-I from membrane-bound organelles at sites of vesicle delivery and membrane insertion. These findings suggest that mutations in PS1 may compromise neuronal function, affecting GSK-3*β* activity and kinesin-I-based motility, thus, leading to neurodegeneration [128].

## 10. Presenilin 2

**Figure 4:**
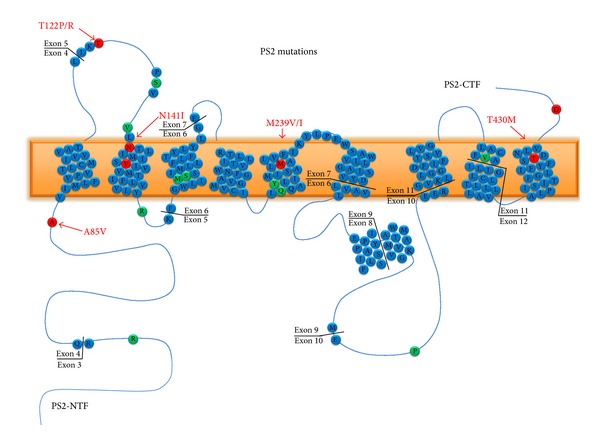
Schematic representation of Presenilin 1. The PSEN2 gene is located on chromosome 1q42.13 and comprises 12 exons, of which only 10 are translated to generate a protein with a length of 448 amino acid residues. This protein exhibits 9 transmembrane domains and displays tissue-specific alternative splicing; major mutations found in the protein are identified.

The PS2 gene is located on chromosome 1q42.13 and comprises 12 exons, only 10 of which are translated to generate a protein with a length of 448 amino acid residues. This protein exhibits 9 transmembrane domains and displays tissue-specific alternative splicing [129] (Figure 4). PS2 mutations are very rare, and only 13 mutations have been described among 22 families (http://www.molgen.ua.ac.be/ADmutations/). In the CNS, PS1 is found mainly in neurons. PS1 is expressed at higher levels during development than PS2, although in the adult brain, PS1 and PS2 are expressed at relatively similar levels and with a similar distribution. Unlike PS1, PS2-knockout mice are viable and exhibit at most a mild pulmonary phenotype [130]. Transgenic mice expressing a mutant form of PS2 (N141I) showed hyperactivity followed by hypoactivity in an open field test as well as lower expression of c-Fos and higher expression of the gamma-aminobutyric acid A receptor subunit alpha 1 in the cortex, hippocampus, and amygdala [131]. PS2 and PS1 may act differently with regard to A*β* generation. Although PS2 shows close homology to PS1, PS2 is less efficient with respect to amyloid peptide production [132]. *In vitro* expression of PS2 V393M cDNA did not result in a detectable increase in the secreted A*β*42/40 peptide ratio. However, patients heterozygous for this missense mutation are characterized by profound language impairment [133].

## 11. Presenilin Mutations and Oxidative Stress

As mentioned above, mutations in PS have been shown to change the processing of APP by altering *γ*-secretase, which in turn lead to higher levels of the amyloidogenic form A*β*. In this sense, it has been shown that the transgenic mouse models expressing AD mutations in PS1 develop mitochondrial abnormalities before cognitive deficits as has been described. In 2006, Schuessel et al. demonstrated that transgenic mice expressed human PS1 with the mutation M146L (PS1M146L), which increases mitochondrial ROS formation as well as oxidative damage in aged mice. They analyzed lipid peroxidation products, such as HNE and malondialdehyde in brain tissue, and levels of ROS in splenic lymphocytes. The results showed that HNE levels increased only in older (19–22-month-old) PS1M146L mice. Similarly in transgenic mice, mitochondrial and cytosolic ROS levels were elevated by 142.1 and 120.5%, respectively. It was also demonstrated that HNE levels of brain tissue were positively correlated with mitochondrial ROS levels in splenic lymphocyte. These results suggest that the combined effect of aging and mutations in PS1 generate oxidative damage that eventually leads to the neurodegenerative process [134]. Oxidative stress is closely linked with mitochondrial abnormalities, which were also reported in PS1M146L transgenic mice, in which caspase activation follows exposure to A*β* peptide and metabolic insults [135].

## 12. Antioxidant Therapy in APP and Presenilin Mutations

Since it has been shown that oxidative stress has an important role in the development of FAD pathology, and its effects can be clearly seen in animal models of this disease, it is important to evaluate whether therapies which target is to reduce oxidative stress have reported to be useful in animal models carrying FAD mutations.

Using Tg2576 mice, Sung et al. demonstrated that vitamin E treatment was able to reduce oxidative stress, LPO, and A*β* burden when the treatment began at age of 5-month-old, but not when the treatment began at age of 14-month-old, again suggesting an early involvement of oxidative stress in this pathology [136]. Similar results were found by Cole and Frautschy in the same mouse model, testing the effects of docosahexanoic acid. Treatment with this antioxidant was able to reduce oxidative stress, dendritic loss, A*β* deposition, and improved cognitive performance in these mice [137]. Contrasting results were found by Siedlak et al. [138] who did not find differences between *α*-lipoic acid-treated Tg2576 mice and placebo-treated mice with respect to A*β* burden and cognitive performance, despite a significant decrease in oxidative stress.

Dragicevic et al. also found an effect of antioxidant therapy in transgenic APP/PS1 mice. They observed that treatment with melatonin in these mice reduced mitochondrial A*β* levels and reestablished mitochondrial respiratory rates and ATP levels in hippocampus, cortex, and striatum [139].

Additionally, Mcmanus et al. reported that treatment with the antioxidant MitoQ (mitoquinone mesylate) was effective in the prevention of cognitive impairment, oxidative stress, A*β* deposition, astrogliosis, synaptic loss, and caspase activation in 3xTg-AD mice, which express the Swedish mutation and also show tau-related pathology as observed in AD patients [140]. These results apparently show a beneficial effect of antioxidant therapy in the treatment of FAD, although it is important to consider that clinical trials performed in LOAD patients have shown only a very modest effect in memory and cognition improvement and disease progression delay. Clinical trials testing the effect of antioxidants specifically in FAD patients have not been conducted yet, to the extent of our knowledge, but considering the amyloidogenic genetic background of this patients and the more aggressive nature of this AD form, the results may be not very promising.

## 13. Conclusions

EOAD is characterized for the presence of mutations in the APP, PS1, and PS2 genes. These mutations confer an increase of A*β* production and its posterior accumulation, which generates a series of molecular events that lead to a neurodegenerative process. Amyloid has the ability to interact with several different receptor types, including the frizzled, insulin, NMDA, and NGF receptors, which trigger events that lead to neuronal death. Most of the transgenic models expressing APP and PS human mutations show high levels of oxidative damage, suggesting that oxidative stress may be an early event in the development of the pathology and has an important role on the fast progression of EOAD compared with LOAD. Moreover, this oxidative damage can increase the synthesis and aggregation of A*β*, which represents a vicious circle that favors peptide toxicity and neurodegeneration. In this sense, it has been suggested as several numbers of therapeutic approaches. The principal strategies include to antioxidants agents, NMDAR antagonists, and the A*β*-immunotherapy. All of these strategies focus on the decrease of A*β* oxidative activity and the toxic effects of aggregates. Therapeutic strategies could delay neurodegeneration, improving the quality of life of EOAD patients for a while, but the genetic background imposes the amyloidosis. In these AD cases, gene therapy may be the best strategy.

## Figures and Tables

**Table 1:** Amyloid precursor protein mutations.

Mutation	Phenotype	Age of onset	References
E665D	AD, but may not be pathogenic		Peacock et al., 1994 [141]
KM670/671NL (Swedish)	AD	52 (44–59)	Mullan et al., 1992 [81]
H677R	AD	55 (55-56)	Janssen et al., 2003 [142]
D678N (Tottori)	FAD	60	Wakutani et al., 2004 [143]
E693Δ	AD		Tomiyama et al., 2008 [144]
D694N (Iowa)	AD or cerebral hemorrhage	69	Grabowski et al., 2001 [83]
A713T	AD, but may not be pathogenic	59	Carter et al., 1992 [145]
T714A (Iranian)	AD	52 (40–60)	Pasalar et al., 2002 [146]
T714I (Austrian)	Affects *γ*-secretase cleavage directly, 11X increase in A*β*(42)/A*β*(40) ratio *in vitro*.		Kumar-Singh et al. [147]
V715A (German)	AD	47	De Jonghe et al., 2001; [148] Cruts et al., 2003 [149]
V715M (French)	AD	52 (40–60)	Ancolio et al., 1999 [150]
I716T	AD	55	Terrini et al., 2002 [151]
I716V (Florida)	AD	55	Eckman et al., 1997 [82]
V717F (Indiana)	AD	47 (42–52)	Murrell et al., 1991 [77]
V717G	AD	55 (45–62)	Chartier-Harlin et al., 1991 [72]
V717I (London)	AD	55 (50–60)	Goate et al., 1991 [74]
T719P	AD	46	Ghidoni et al., 2009 [152]
L723P (Australian)	AD	56 (45–60)	Kwok et al., 2000 [153]

## References

[1] Gilgun-Sherki Y, Melamed E, Offen D (2001). Oxidative stress induced-neurodegenerative diseases: the need for antioxidants that penetrate the blood brain barrier. *Neuropharmacology*.

[2] Velayutham M, Hemann C, Zweier JL (2011). Removal of H_2_O_2_ and generation of superoxide radical: role of cytochrome c and NADH. *Free Radical Biology and Medicine*.

[3] Uttara B, Singh AV, Zamboni P, Mahajan RT (2009). Oxidative stress and neurodegenerative diseases: a review of upstream and downstream antioxidant therapeutic options. *Current Neuropharmacology*.

[4] Nemoto S, Takeda K, Yu Z-X, Ferrans VJ, Finkel T (2000). Role for mitochondrial oxidants as regulators of cellular metabolism. *Molecular and Cellular Biology*.

[5] Floyd RA, Carney JM (1992). Free radical damage to protein and DNA: mechanisms involved and relevant observations on brain undergoing oxidative stress. *Annals of Neurology*.

[6] Shohami E, Beit-Yannai E, Horowitz M, Kohen R (1997). Oxidative stress in closed-head injury: brain antioxidant capacity as an indicator of functional outcome. *Journal of Cerebral Blood Flow and Metabolism*.

[7] Jones DP (2008). Radical-free biology of oxidative stress. *American Journal of Physiology*.

[8] Nguyen D, Alavi MV, Kim K-Y (2011). A new vicious cycle involving glutamate excitotoxicity, oxidative stress and mitochondrial dynamics. *Cell Death and Disease*.

[9] Federico A, Cardaioli E, Da Pozzo P, Formichi P, Gallus G, Radi E (2012). Mitochondria, oxidative stress and neurodegeneration. *Journal of the Neurological Sciences*.

[10] McKhann G, Drachman D, Folstein M (1984). Clinical diagnosis of Alzheimer’s disease: report of the NINCDS-ADRDA work group under the auspices of Department of Health and Human Services Task Force on Alzheimer’s disease. *Neurology*.

[11] Mukaetova-Ladinska EB, Harrington CR, Roth M, Wischik CM (1993). Biochemical and anatomical redistribution of tau protein in Alzheimer’s disease. *American Journal of Pathology*.

[12] Fukuchi K, Hart M, Li L (1998). Alzheimer’s disease and heparan sulfate proteoglycan. *Frontiers in Bioscience*.

[13] Bergem AL, Engedal K, Kringlen E (1997). The role of heredity in late-onset Alzheimer disease and vascular dementia: a twin study. *Archives of General Psychiatry*.

[14] Gatz M, Reynolds CA, Fratiglioni L (2006). Role of genes and environments for explaining Alzheimer disease. *Archives of General Psychiatry*.

[15] Räihä I, Kaprio J, Koskenvuo M, Rajala T, Sourander L (1996). Alzheimer’s disease in Finnish twins. *The Lancet*.

[16] Aleshkov S, Abraham CR, Zannis VI (1997). Interaction of nascent apoe2, apoe3, and apoe4 isoforms expressed in mammalian cells with amyloid peptide *β* (1-40). Relevance to Alzheimer’s disease. *Biochemistry*.

[17] Corder EH, Saunders AM, Strittmatter WJ (1993). Gene dose of apolipoprotein E type 4 allele and the risk of Alzheimer’s disease in late onset families. *Science*.

[18] Saunders AM, Strittmatter WJ, Schmechel D (1993). Association of apolipoprotein E allele *ε*4 with late-onset familial and sporadic Alzheimer’s disease. *Neurology*.

[19] Strittmatter WJ, Roses AD (1995). Apolipoprotein E and Alzheimer disease. *Proceedings of the National Academy of Sciences of the United States of America*.

[20] Ertekin-Taner N (2007). Genetics of Alzheimer’s disease: a centennial review. *Neurologic Clinics*.

[21] Kim J, Basak JM, Holtzman DM (2009). The Role of Apolipoprotein E in Alzheimer’s Disease. *Neuron*.

[22] Roberts GW, Nash M, Ince PG, Royston MC, Gentleman SM (1993). On the origin of Alzheimer’s disease: a hypothesis. *NeuroReport*.

[23] Terry RD, Masliah E, Salmon DP (1991). Physical basis of cognitive alterations in Alzheimer’s disease: synapse loss is the major correlate of cognitive impairment. *Annals of Neurology*.

[24] Tomlinson BEC, Hume Adams JC, Duchen LW (1984). Ageing and the dementias. *Greenfield's Neuropathologyed*.

[25] Blessed G, Tomlinson BE, Roth M (1968). The association between quantitative measures of dementia and of senile change in the cerebral grey matter of elderly subjects. *British Journal of Psychiatry*.

[26] Braak H, Braak E (1997). Staging of Alzheimer-related cortical destruction. *International Psychogeriatrics*.

[27] Andreadis A, Brown WM, Kosik KS (1992). Structure and novel exons of the human *τ* gene. *Biochemistry*.

[28] Ennulat DJ, Liem RKH, Hashim GA, Shelanski ML (1989). Two separate 18-amino acid domains of tau promote the polymerization of tubulin. *Journal of Biological Chemistry*.

[29] Goedert M, Spillantini MG, Jakes R, Rutherford D, Crowther RA (1989). Multiple isoforms of human microtubule-associated protein tau: sequences and localization in neurofibrillary tangles of Alzheimer’s disease. *Neuron*.

[30] Goedert M, Wischik CM, Crowther RA, Walker JE, Klug A (1988). Cloning and sequencing of the cDNA encoding a core protein of the paired helical filament of Alzheimer disease: identification as the microtubule-associated protein tau. *Proceedings of the National Academy of Sciences of the United States of America*.

[31] Cleveland DW, Hwo SY, Kirschner MW (1977). Physical and chemical properties of purified tau factor and the role of tau in microtubule assembly. *Journal of Molecular Biology*.

[32] Schweers O, Schönbrunn-Hanebeck E, Marx A, Mandelkow E (1994). Structural studies of tau protein and Alzheimer paired helical filaments show no evidence for *β*-structure. *Journal of Biological Chemistry*.

[33] Goedert M, Klug A (1999). Tau protein and the paired helical filament of Alzheimer’s disease. *Brain Research Bulletin*.

[34] Ballatore C, Lee VM-Y, Trojanowski JQ (2007). Tau-mediated neurodegeneration in Alzheimer’s disease and related disorders. *Nature Reviews Neuroscience*.

[35] Chen F, David D, Ferrari A, Götz J (2004). Posttranslational modifications of tau—role in human tauopathies and modeling in transgenic animals. *Current Drug Targets*.

[36] Hernández F, Avila J (2007). Tauopathies. *Cellular and Molecular Life Sciences*.

[37] Hutton M (2000). Molecular genetics of chromosome 17 tauopathies. *Annals of the New York Academy of Sciences*.

[38] Hutton M, Lendon CL, Rizzu P (1998). Association of missense and 5’-splice-site mutations in tau with the inherited dementia FTDP-17. *Nature*.

[39] Poorkaj P, Bird T, Wijsman E (1998). Tau is a candidate gene for chromosome 17 frontotemporal dementia. *Annals of Neurology*.

[40] Spillantini MG, Bird TD, Ghetti B (1998). Frontotemporal dementia and Parkinsonism linked to chromosome 17: a new group of tauopathies. *Brain Pathology*.

[41] Iversen LL, Mortishire-Smith RJ, Pollack SJ, Shearman MS (1995). The toxicity in vitro of *β*-amyloid protein. *Biochemical Journal*.

[42] Glenner GG, Wong CW, Quaranta V, Eanes ED (1984). The amyloid deposits in Alzheimer’s disease: their nature and pathogenesis. *Applied Pathology*.

[43] Selkoe DJ (1994). Alzheimer’s disease: a central role for amyloid. *Journal of Neuropathology and Experimental Neurology*.

[44] Kang J, Lemaire H-G, Unterbeck A (1987). The precursor of Alzheimer’s disease amyloid A4 protein resembles a cell-surface receptor. *Nature*.

[45] Schmitz A, Tikkanen R, Kirfel G, Herzog V (2002). The biological role of the Alzheimer amyloid precursor protein in epithelial cells. *Histochemistry and Cell Biology*.

[46] Schubert D, Jin L, Saitoh T, Cole G (1989). The regulation of amyloid *β* protein precursor secretion and its modulatory role in cell adhesion. *Neuron*.

[47] Crowther RA, Wischik CM (1985). Image reconstruction of the Alzheimer paired helical filament. *The EMBO Journal B*.

[48] Turner PR, O’Connor K, Tate WP, Abraham WC (2003). Roles of amyloid precursor protein and its fragments in regulating neural activity, plasticity and memory. *Progress in Neurobiology*.

[49] Priller C, Bauer T, Mitteregger G, Krebs B, Kretzschmar HA, Herms J (2006). Synapse formation and function is modulated by the amyloid precursor protein. *The Journal of Neuroscience*.

[50] Kimberly WT, LaVoie MJ, Ostaszewski BL, Ye W, Wolfe MS, Selkoe DJ (2003). *γ*-Secretase is a membrane protein complex comprised of presenilin, nicastrin, aph-1, and pen-2. *Proceedings of the National Academy of Sciences of the United States of America*.

[51] Li T, Ma G, Cai H, Price DL, Wong PC (2003). Nicastrin is required for assembly of presenilin/*γ*-secretase complexes to mediate notch signaling and for processing and trafficking of *β*-amyloid precursor protein in mammals. *Journal of Neuroscience*.

[52] Li Y, Lai M, Xu M (2000). Presenilin 1 is linked with *γ*-secretase activity in the detergent solubilized state. *Proceedings of the National Academy of Sciences of the United States of America*.

[53] Yu G, Nishimura M, Arawaka S (2000). Nicastrin modulates presenilin-mediated notch/glp-1 signal transduction and *βAPP* processing. *Nature*.

[54] Sakono M, Zako T (2010). Amyloid oligomers: formation and toxicity of A*β* oligomers. *FEBS Journal*.

[55] Kulic L, McAfoose J, Welt T (2012). Early accumulation of intracellular fibrillar oligomers and late congophilic amyloid angiopathy in mice expressing the Osaka intra-Abeta *APP* mutation. *Translational Psychiatry*.

[56] Benilova I, Karran E, De Strooper B (2012). The toxic A*β* oligomer and Alzheimer’s disease: an emperor in need of clothes. *Nature Neuroscience*.

[57] Deibel MA, Ehmann WD, Markesbery WR (1996). Copper, iron, and zinc imbalances in severely degenerated brain regions in Alzheimer’s disease: possible relation to oxidative stress. *Journal of the Neurological Sciences*.

[58] Opazo C, Huang X, Cherny RA (2002). Metalloenzyme-like activity of Alzheimer’s disease *β*-amyloid: Cu-dependent catalytic conversion of dopamine, cholesterol, and biological reducing agents to neurotoxic H_2_O_2_. *Journal of Biological Chemistry*.

[59] Jiang D, Li X, Williams R (2009). Ternary complexes of iron, amyloid-*β*, and nitrilotriacetic acid: binding affinities, redox properties, and relevance to iron-induced oxidative stress in Alzheimer’s disease. *Biochemistry*.

[60] Cuajungco MP, Goldstein LE, Nunomura A (2000). Evidence that the *β*-amyloid plaques of Alzheimer’s disease represent the redox-silencing and entombment of A*β* by zinc. *Journal of Biological Chemistry*.

[61] Yatin SM, Varadarajan S, Link CD, Butterfield DA (1999). In vitro and in vivo oxidative stress associated with Alzheimer’s amyloid *β*-peptide (1–42). *Neurobiology of Aging*.

[62] Butterfield DA, Galvan V, Lange MB (2010). In vivo oxidative stress in brain of Alzheimer disease transgenic mice: requirement for methionine 35 in amyloid *β*-peptide of *APP*. *Free Radical Biology and Medicine*.

[63] Barnham KJ, Ciccotosto GD, Tickler AK (2003). Neurotoxic, redox-competent Alzheimer’s *β*-amyloid is released from lipid membrane by methionine oxidation. *Journal of Biological Chemistry*.

[64] Moskovitz J, Maiti P, Lopes DHJ (2011). Induction of methionine-sulfoxide reductases protects neurons from amyloid *β*-protein insults in vitro and in vivo. *Biochemistry*.

[65] Meraz-Rios MA, Toral-Rios D, Franco-Bocanegra D, Villeda-Hernandez J, Campos-Pena V (2013). Inflammatory process in Alzheimer's disease. *Frontiers in Integrative Neuroscience*.

[66] De Felice FG, Velasco PT, Lambert MP (2007). A*β* oligomers induce neuronal oxidative stress through an N-methyl-D-aspartate receptor-dependent mechanism that is blocked by the Alzheimer drug memantine. *Journal of Biological Chemistry*.

[67] Shelat PB, Chalimoniuk M, Wang J (2008). Amyloid beta peptide and NMDA induce ROS from NADPH oxidase and AA release from cytosolic phospholipase A2 in cortical neurons. *Journal of Neurochemistry*.

[68] Cha MY, Han S, Son SM (2012). Mitochondria-specific accumulation of amyloid *β* induces mitochondrial dysfunction leading to apoptotic cell death. *PLoS ONE*.

[69] Abramov AY, Canevari L, Duchen MR (2004). *β*-amyloid peptides induce mitochondrial dysfunction and oxidative stress in astrocytes and death of neurons through activation of NADPH oxidase. *Journal of Neuroscience*.

[70] Buttini M, Masliah E, Barbour R (2005). *β*-amyloid immunotherapy prevents synaptic degeneration in a mouse model of Alzheimer’s disease. *Journal of Neuroscience*.

[71] Borchelt DR, Thinakaran G, Eckman CB (1996). Familial Alzheimer’s disease-linked presenilin I variants elevate a*β*1- 42/1-40 ratio in vitro and in vivo. *Neuron*.

[72] Chartier-Harlin MC, Crawford F, Hamandi K (1991). Screening for the *β*-amyloid precursor protein mutation (*APP*717: Val → Ile) in extended pedigrees with early onset Alzheimer’s disease. *Neuroscience Letters*.

[73] Duff K, Eckman C, Zehr C (1996). Increased amyloid-*β*42(43) in brains of mice expressing mutant presenilin 1. *Nature*.

[74] Goate A, Chartier-Harlin M-C, Mullan M (1991). Segregation of a missense mutation in the amyloid precursor protein gene with familial Alzheimer’s disease. *Nature*.

[75] Kovacs DM, Fausett HJ, Page KJ (1996). Alzheimer-associated presenilins 1 and 2: neuronal expression in brain and localization to intracellular membranes in mammalian cells. *Nature Medicine*.

[76] Levy-Lahad E, Wijsman EM, Nemens E (1995). A familial Alzheimer’s disease locus on chromosome I. *Science*.

[77] Murrell J, Farlow M, Ghetti B, Benson MD (1991). A mutation in the amyloid precursor protein associated with hereditary Alzheimer’s disease. *Science*.

[78] Scheuner D, Eckman C, Jensen M (1996). Secreted amyloid *β*-protein similar to that in the senile plaques of Alzheimer’s disease is increased in vivo by the presenilin 1 and 2 and *APP* mutations linked to familial Alzheimer’s disease. *Nature Medicine*.

[79] Sisodia SS, Kim SH, Thinakaran G (1999). Function and dysfunction of the presenilins. *American Journal of Human Genetics*.

[80] Levy E, Carman MD, Fernandez-Madrid IJ (1990). Mutation of the Alzheimer’s disease amyloid gene in hereditary cerebral hemorrhage, Dutch type. *Science*.

[81] Mullan M, Crawford F, Axelman K (1992). A pathogenic mutation for probable Alzheimer’s disease in the *APP* gene at the N-terminus of *β*-amyloid. *Nature Genetics*.

[82] Eckman CB, Mehta ND, Crook R (1997). A new pathogenic mutation in the *APP* gene (1716V) increases the relative proportion of A*β*42(43). *Human Molecular Genetics*.

[83] Grabowski TJ, Cho HS, Vonsattel JPG, William Rebeck G, Greenberg SM (2001). Novel amyloid precursor protein mutation in an Iowa family with dementia and severe cerebral amyloid angiopathy. *Annals of Neurology*.

[84] Nilsberth C, Westlind-Danielsson A, Eckman CB (2001). The “Arctic” *APP* mutation (E693G) causes Alzheimer’s disease by enhanced A*β* protofibril formation. *Nature Neuroscience*.

[85] Nunan J, Small DH (2000). Regulation of *APP* cleavage by *α*-, *β*- and *γ*-secretases. *FEBS Letters*.

[86] Perez RG, Squazzo SL, Koo EH (1996). Enhanced release of amyloid *β*-protein from codon 670/671 “Swedish” mutant *β*-amyloid precursor protein occurs in both secretory and endocytic pathways. *Journal of Biological Chemistry*.

[87] Du H, Guo L, Yan S, Sosunov AA, McKhann GM, Yan SS (2010). Early deficits in synaptic mitochondria in an Alzheimer’s disease mouse model. *Proceedings of the National Academy of Sciences of the United States of America*.

[88] Bobba A, Amadoro G, Valenti D, Corsetti V, Lassandro R, Atlante A (2013). Mitochondrial respiratory chain complexes I and IV are impaired by beta-amyloid via direct interaction and through complex I-dependent ROS production, respectively. *Mitochondrion*.

[89] Ren R, Zhang Y, Lee B, Wu Y, Li B (2011). Effect of *β*-amyloid (25–35) on mitochondrial function and expression of mitochondrial permeability transition pore proteins in rat hippocampal neurons. *Journal of Cellular Biochemistry*.

[90] Manczak M, Calkins MJ, Reddy PH (2011). Impaired mitochondrial dynamics and abnormal interaction of amyloid beta with mitochondrial protein Drp1 in neurons from patients with Alzheimer’s disease: implications for neuronal damage. *Human Molecular Genetics*.

[91] Jungas T, Motta I, Duffieux F, Fanen P, Stoven V, Ojcius DM (2002). Glutathione levels and BAX activation during apoptosis due to oxidative stress in cells expressing wild-type and mutant cystic fibrosis transmembrane conductance regulator. *Journal of Biological Chemistry*.

[92] Hauptmann S, Scherping I, Dröse S (2009). Mitochondrial dysfunction: an early event in Alzheimer pathology accumulates with age in AD transgenic mice. *Neurobiology of Aging*.

[93] Praticò D, Uryu K, Leight S, Trojanoswki JQ, Lee VM-Y (2001). Increased lipid peroxidation precedes amyloid plaque formation in an animal model of alzheimer amyloidosis. *Journal of Neuroscience*.

[94] Butterfield DA, Bader Lange ML, Sultana R (2010). Involvements of the lipid peroxidation product, HNE, in the pathogenesis and progression of Alzheimer’s disease. *Biochimica et Biophysica Acta*.

[95] Apelt J, Bigl M, Wunderlich P, Schliebs R (2004). Aging-related increase in oxidative stress correlates with developmental pattern of beta-secretase activity and beta-amyloid plaque formation in transgenic Tg2576 mice with Alzheimer-like pathology. *International Journal of Developmental Neuroscience*.

[96] Manczak M, Anekonda TS, Henson E, Park BS, Quinn J, Reddy PH (2006). Mitochondria are a direct site of A*β* accumulation in Alzheimer’s disease neurons: implications for free radical generation and oxidative damage in disease progression. *Human Molecular Genetics*.

[97] Blanchard V, Moussaoui S, Czech C (2003). Time sequence of maturation of dystrophic neurites associated with A*β* deposits in *APP*/*PS1* transgenic mice. *Experimental Neurology*.

[98] Schuessel K, Schäfer S, Bayer TA (2005). Impaired Cu/Zn-SOD activity contributes to increased oxidative damage in *APP* transgenic mice. *Neurobiology of Disease*.

[99] Sherrington R, Froelich S, Sorbi S (1996). Alzheimer’s disease associated with mutations in presenilin 2 is rare and variably penetrant. *Human Molecular Genetics*.

[100] Chyung JH, Raper DM, Selkoe DJ (2005). *γ*-secretase exists on the plasma membrane as an intact complex that accepts substrates and effects intramembrane cleavage. *Journal of Biological Chemistry*.

[101] Réchards M, Xia W, Oorschot VMJ, Selkoe DJ, Klumperman J (2003). Presenilin-1 exist in both pre- and post-golgi compartments and recycles via COPI-coated membranes. *Traffic*.

[102] Vetrivel KS, Cheng H, Lin W (2004). Association of *γ*-secretase with lipid rafts in post-golgi and endosome membranes. *Journal of Biological Chemistry*.

[103] Ebinu JO, Yankner BA (2002). A RIP tide in neuronal signal transduction. *Neuron*.

[104] Kulic L, Walter J, Multhaup G (2000). Separation of presenilin function in amyloid *β*-peptide generation and endoproteolysis of Notch. *Proceedings of the National Academy of Sciences of the United States of America*.

[105] Marambaud P, Shioi J, Serban G (2002). A presenilin-1/*γ*-secretase cleavage releases the E-cadherin intracellular domain and regulates disassembly of adherens junctions. *EMBO Journal*.

[106] Vassar R, Citron M (2000). A*β*-generating enzymes: recent advances in *β*- and *γ*-secretase research. *Neuron*.

[107] Yu JT, Song J, Ma T (2011). Genetic association of PICALM polymorphisms with Alzheimer’s disease in Han Chinese. *Journal of the Neurological Sciences*.

[108] Naruse S, Thinakaran G, Luo J (1998). Effects of *PS1* deficiency on membrane protein trafficking in neurons. *Neuron*.

[109] Pratt KG, Zimmerman EC, Cook DG, Sullivan JM (2011). Presenilin 1 regulates homeostatic synaptic scaling through Akt signaling. *Nature Neuroscience*.

[110] Saura CA, Servián-Morilla E, Scholl FG (2011). Presenilin/*γ*-secretase regulates neurexin processing at synapses. *PLoS ONE*.

[111] Shimizu T, Toda T, Noda Y, Ito G, Maeda M (2011). Presenilin-2 mutation causes early amyloid accumulation and memory impairment in a transgenic mouse model of Alzheimer’s disease. *Journal of Biomedicine and Biotechnology*.

[112] Supnet C, Bezprozvanny I (2011). Presenilins function in ER calcium leak and Alzheimer’s disease pathogenesis. *Cell Calcium*.

[113] Zhang H, Sun S, Herreman A, De Strooper B, Bezprozvanny I (2010). Role of presenilins in neuronal calcium homeostasis. *Journal of Neuroscience*.

[114] Zhang C, Wu B, Beglopoulos V (2009). Presenilins are essential for regulating neurotransmitter release. *Nature*.

[115] Louvi A, Sisodia SS, Grove EA (2004). Presenilin 1 in migration and morphogenesis in the central nervous system. *Development*.

[116] Busciglio J, Hartmann H, Lorenzo A (1997). Neuronal localization of presenilin-1 and association with amyloid plaques and neurofibrillary tangles in Alzheimer’s disease. *Journal of Neuroscience*.

[117] Jacobsen H, Reinhardt D, Brockhaus M (1999). The influence of endoproteolytic processing of familial Alzheimer’s disease presenilin 2 on A*β*42 amyloid peptide formation. *Journal of Biological Chemistry*.

[118] Storey E, Cappai R (1999). The amyloid precursor protein of Alzheimer’s disease and the A*β* peptide. *Neuropathology and Applied Neurobiology*.

[119] Wolfe MS, De Los Angeles J, Miller DD, Xia W, Selkoe DJ (1999). Are presenilins intramembrane-cleaving proteases? Implications for the molecular mechanism of Alzheimer’s disease. *Biochemistry*.

[120] Wolfe MS, Xia W, Ostaszewski BL, Diehl TS, Kimberly WT, Selkoe DJ (1999). Two transmembrane aspartates in presenilin-1 required for presenilin endoproteolysis and *γ*-secretase activity. *Nature*.

[121] Wisniewski T, Dowjat WK, Buxbaum JD (1998). A novel polish presenilin-1 mutation (P117L) is associated with familial Alzheimer’s disease and leads to death as early as the age of 28 years. *NeuroReport*.

[122] Campion D, Brice A, Dumanchin C (1996). A novel presenilin 1 mutation resulting in familial Alzheimer’s disease with an onset age of 29 years. *NeuroReport*.

[123] Shen J, Bronson RT, Chen DF, Xia W, Selkoe DJ, Tonegawa S (1997). Skeletal and CNS defects in Presenilin-1-deficient mice. *Cell*.

[124] Citron M, Westaway D, Xia W (1997). Mutant presenilins of Alzheimer’s disease increase production of 42-residue amyloid *β*-protein in both transfected cells and transgenic mice. *Nature Medicine*.

[125] Casas C, Sergeant N, Itier J (2004). Massive CA1/2 neuronal loss with intraneuronal and N-terminal truncated A*β*42 accumulation in a novel Alzheimer transgenic model. *American Journal of Pathology*.

[126] Christensen DZ, Kraus SL, Flohr A, Cotel M, Wirths O, Bayer TA (2008). Transient intraneuronal A*β* rather than extracellular plaque pathology correlates with neuron loss in the frontal cortex of *APP*/*PS1*KI mice. *Acta Neuropathologica*.

[127] Breyhan H, Wirths O, Duan K, Marcello A, Rettig J, Bayer TA (2009). *APP*/*PS1*KI bigenic mice develop early synaptic deficits and hippocampus atrophy. *Acta Neuropathologica*.

[128] Pigino G, Morfini G, Pelsman A, Mattson MP, Brady ST, Busciglio J (2003). Alzheimer’s presenilin 1 mutations impair kinesin-based axonal transport. *Journal of Neuroscience*.

[129] Prihar G, Fuldner RA, Perez-Tur J (1996). Structure and alternative splicing of the presenilin-2 gene. *NeuroReport*.

[130] Herreman A, Hartmann D, Annaert W (1999). Presenilin 2 deficiency causes a mild pulmonary phenotype and no changes in amyloid precursor protein processing but enhances the embryonic lethal phenotype of presenilin 1 deficiency. *Proceedings of the National Academy of Sciences of the United States of America*.

[131] Yuk DY, Lee YK, Nam SY (2009). Reduced anxiety in the mice expressing mutant (N141I) presenilin 2. *Journal of Neuroscience Research*.

[132] Bentahir M, Nyabi O, Verhamme J (2006). Presenilin clinical mutations can affect *γ*-secretase activity by different mechanisms. *Journal of Neurochemistry*.

[133] Lindquist SG, Hasholt L, Bahl JMC (2008). A novel presenilin 2 mutation (V393M) in early-onset dementia with profound language impairment. *European Journal of Neurology*.

[134] Schuessel K, Frey C, Jourdan C (2006). Aging sensitizes toward ROS formation and lipid peroxidation in *PS1*M146L transgenic mice. *Free Radical Biology and Medicine*.

[135] Begley JG, Duan W, Chan S, Duff K, Mattson MP (1999). Altered calcium homeostasis and mitochondrial dysfunction in cortical synaptic compartments of presenilin-1 mutant mice. *Journal of Neurochemistry*.

[136] Sung S, Yao Y, Uryu K (2004). Early vitamin E supplementation in young but not aged mice reduces Abeta levels and amyloid deposition in a transgenic model of Alzheimer’s disease. *The FASEB Journal*.

[137] Cole GM, Frautschy SA (2006). Docosahexaenoic acid protects from amyloid and dendritic pathology in an Alzheimer’s disease mouse model. *Nutrition and Health*.

[138] Siedlak SL, Casadesus G, Webber KM (2009). Chronic antioxidant therapy reduces oxidative stress in a mouse model of Alzheimer’s disease. *Free Radical Research*.

[139] Dragicevic N, Copes N, O’Neal-Moffitt G (2011). Melatonin treatment restores mitochondrial function in Alzheimer’s mice: a mitochondrial protective role of melatonin membrane receptor signaling. *Journal of Pineal Research*.

[140] Mcmanus MJ, Murphy MP, Franklin JL (2011). The mitochondria-targeted antioxidant mitoQ prevents loss of spatial memory retention and early neuropathology in a transgenic mouse model of Alzheimer’s disease. *Journal of Neuroscience*.

[141] Peacock ML, Murman DL, Sima AAF, Warren JT, Roses AD, Fink JK (1994). Novel amyloid precursor protein gene mutation (codon 665(Asp)) in a patient with late-onset Alzheimer’s disease. *Annals of Neurology*.

[142] Janssen JC, Beck JA, Campbell TA (2003). Early onset familial Alzheimer’s disease: mutation frequency in 31 families. *Neurology*.

[143] Wakutani Y, Watanabe K, Adachi Y (2004). Novel amyloid precursor protein gene missense mutation (D678N) in probable familial Alzheimer’s disease. *Journal of Neurology, Neurosurgery and Psychiatry*.

[144] Tomiyama T, Nagata T, Shimada H (2008). A new amyloid *β* variant favoring oligomerization in Alzheimer’s-type dementia. *Annals of Neurology*.

[145] Carter DA, Desmarais E, Bellis M (1992). More missense in amyloid gene. *Nature Genetics*.

[146] Pasalar P, Najmabadi H, Noorian AR (2002). An Iranian family with Alzheimer’s disease caused by a novel APP mutation (THr714ALa). *Neurology*.

[147] Kumar-Singh S, De Jonghe C, Cruts M (2000). Nonfibrillar diffuse amyloid deposition due to a *γ*42-secretase site mutation points to an essential role for N-truncated A*β*42 in Alzheimer’s disease. *Human Molecular Genetics*.

[148] De Jonghe C, Esselens C, Kumar-Singh S (2001). Pathogenic APP mutations near the *γ*-secretase cleavage site differentially affect A*β* secretion and APP C-terminal fragment stability. *Human Molecular Genetics*.

[149] Cruts M, Dermaut B, Rademakers R, Van Den Broeck M, Stögbauer F, Van Broeckhoven C (2003). Novel APP mutation V715A associated with presenile Alzheimer’s disease in a German family. *Journal of Neurology*.

[150] Ancolio K, Dumanchin C, Barelli H (1999). Unusual phenotypic alteration of *β* amyloid precursor protein (*β*APP) maturation by a new Val-715 → Met *β*APP-770 mutation responsible for probable early-onset Alzheimer’s disease. *Proceedings of the National Academy of Sciences of the United States of America*.

[151] Terreni L, Fogliarino S, Franceschi, Forloni G (2002). Novel pathogenic mutation in an Italian patient with familial Alzheimer's disease detected in APP gene. *Neurobiology of Aging*.

[152] Ghidoni R, Albertini V, Squitti R (2009). Novel T719P A*β*PP mutation unbalances the relative proportion of amyloid-*β* peptides. *Journal of Alzheimer’s Disease*.

[153] Kwok JB, Li QX, Hallupp M (2000). Novel Leu723Pro amyloid precursor protein mutation increases amyloid *β*42(43) peptide levels and induces apoptosis. *Annals of Neurology*.

